# Red blood cell indices and anaemia as causative factors for cognitive function deficits and for Alzheimer’s disease

**DOI:** 10.1186/s13073-018-0556-z

**Published:** 2018-06-28

**Authors:** Laura M. Winchester, John Powell, Simon Lovestone, Alejo J. Nevado-Holgado

**Affiliations:** 1 Department of Psychiatry, University of Oxford, Oxford, UK; 2 Institute of Psychiatry, Psychology and Neuroscience, Kings College London, London, UK

**Keywords:** Anaemia, Alzheimer’s disease, Cognitive function, Mendelian randomisation

## Abstract

**Background:**

Studies have shown that low haemoglobin and anaemia are associated with poor cognition, and anaemia is known to be associated with Alzheimer’s disease (AD), but the mechanism of this risk is unknown. Here, we first seek to confirm the association between cognition and anaemia and secondly, in order to further understand the mechanism of this association, to estimate the direction of causation using Mendelian randomisation.

**Methods:**

Two independent cohorts were used in this analysis: AddNeuroMed, a longitudinal study of 738 subjects including AD and age-matched controls with blood cell measures, cognitive assessments and gene expression data from blood; and UK Biobank, a study of 502,649 healthy participants, aged 40–69 years with cognitive test measures and blood cell indices at baseline. General linear models were calculated using cognitive function as the outcome with correction for age, sex and education. In UK Biobank, SNPs with known blood cell measure associations were analysed with Mendelian randomisation to estimate direction of causality. In AddNeuroMed, gene expression data was used in pathway enrichment analysis to identify associations reflecting biological function.

**Results:**

Both sample sets evidence a reproducible association between cognitive performance and mean corpuscular haemoglobin (MCH), a measure of average mass of haemoglobin per red blood cell. Furthermore, in the AddNeuroMed cohort, where longitudinal samples were available, we showed a greater decline in red blood cell indices for AD patients when compared to controls (*p* values between 0.05 and 10^−6^). In the UK Biobank cohort, we found lower haemoglobin in participants with reduced cognitive function. There was a significant association for MCH and red blood cell distribution width (RDW, a measure of cell volume variability) compared to four cognitive function tests including reaction time and reasoning (*p* < 0.0001). Using Mendelian randomisation, we then showed a significant effect of MCH on the verbal–numeric and numeric traits, implying that anaemia has causative effect on cognitive performance.

**Conclusions:**

Lower haemoglobin levels in blood are associated to poor cognitive function and AD. We have used UK Biobank SNP data to determine the relationship between cognitive testing and haemoglobin measures and suggest that haemoglobin level and therefore anaemia does have a primary causal impact on cognitive performance.

**Electronic supplementary material:**

The online version of this article (10.1186/s13073-018-0556-z) contains supplementary material, which is available to authorized users.

## Background

Dementia, a syndrome increasingly common in our ageing societies, is widely recognised as one of the world’s largest unmet medical needs. Significant progress has been made in identifying the determinative genes of familial diseases that cause dementia, such as early-onset Alzheimer’s disease (AD) or fronto-temporal dementia [1, 2]. For the commonest form of dementia, late-onset AD, genome-wide association studies have identified genes that alter the risk of suffering from the condition [3]. The identification of these genetic factors has driven much of our understanding with respect to the mechanisms of neurodegenerative disease. However, although modifiable environmental factors have also been identified (reviewed in [4]), the role of environmental influences such as cardiovascular risk, depression and social isolation in the disease process is less certain. Most significantly, factors associated with diseases, such as depression and social isolation, could plausibly be consequences, or even prodromal symptoms [5], rather than causes of dementia. It has been suggested that metabolic dysfunction plays a mechanistic role in disease [6] and could be a consequence of the genetically driven molecular pathological process rather than its cause [7, 8]. Clearly, this makes a difference when considering potential interventions to identify or prevent AD.

Another potentially modifiable risk factor for poor cognition in late life is anaemia. Systematic reviews suggest that anaemia is a risk factor for both dementia and for cognitive impairment [9, 10]. In addition to these, Faux et al. [11] found lower haemoglobin and differences in blood measures for mean cell haemoglobin, packed cell volume and higher erythrocyte sedimentation rates in people with AD, while Ferrer et al. [12] found that levels of neuronal haemoglobin are reduced in AD. In the Rush Memory and Aging Project, both high and low levels of haemoglobin were associated with AD and faster cognitive decline [13]. In participants at post-mortem analyses, lower haemoglobin levels were associated with macroscopic infarcts but not other pathologies of neurodegeneration [14]. Although it is reasonably clear that there is a relationship between indices of red blood cell phenotypes and cognition, the directionality and therefore causality of the observation is unknown, just as it is for other environmental factors.

Determining whether potentially modifiable factors associated with dementia are drivers of disease process and hence targets for therapy is of critical importance. A powerful approach to determining such causality is the use of Mendelian randomisation (MR). One of the limitations of MR, however, is the availability of genetic loci strongly associating with the phenotype under consideration. Here, we have utilised growing understanding of the genetic determinants of red blood cell characteristics to explore the role of haemoglobin and anaemia as a causal factor of cognitive phenotypes, including dementia, while integrating this growing understanding with modern MR methods able to combine multiple genetic loci.

We use a range of analyses to draw inferences about the relationship of red blood cell indices, and therefore anaemia, to both cognitive function and AD. Using both UK Biobank and AddNeuroMed data, we confirm the relationship between AD and anaemia. Then, MR methods suggest that altered red blood cell indices are causally associated with reduced cognitive function and finally, we provide transcriptomic evidence for molecular pathways that might underpin this mechanism.

## Methods

### Clinical measures and blood indices

#### UK Biobank

The UK Biobank study is comprised of 502,649 healthy participants, aged 40–69 years with comprehensive phenotypic measures including cognitive testing and blood cell indices (Additional file 1: Table S1), with measures described in detail online [15]. Briefly, blood cell indices were calculated for participants using a haemotology analyser which generated complete count data, including red blood cell count (RBC) and haemoglobin concentration (HGB). Other parameters were calculated from these same measures, e.g. mean corpuscular haemoglobin (MCH). All indices used in this analysis were taken from the recruitment/baseline visit. Anaemia classification was based on NICE guidelines, specifically males with HGB below 13 g/100 mL and women with HGB below 12 g/100 mL.

Results from tests conducted at baseline were used to measure cognitive function. Full assessment methods are described by Lyall et al. [16] but a brief description of cognitive function test and value treatment follows:

##### Verbal–numeric reasoning (fluid intelligence)

13 logic-based questions asked within a 2 minute time limit. Total number of correct responses was used for analysis (UKB Field Identifier (FID) 20016).

##### Numeric memory

Participants were asked to remember a two-digit number after a brief pause. The number of digits was then increased and longest number of digits recalled was used for analysis (FID: 4282).

##### Reaction time

Time taken for participants to match two identical symbols and press button. Mean reaction time (ms) of eight trials was used for analysis after log transformation (FID: 20023).

##### Visual memory

Pair matching test based on memory of card location. Number of pairs mismatched for the six pair test was used for analysis after log transformation (FID: 399).

##### Prospective memory

An instruction was given at the beginning of the assessment, which the participant needs to remember in order to select the correct shape at the end of the interview. A binary success or fail measure of the first attempt was used for further analysis (FID: 20018).

#### AddNeuroMed

AddNeuroMed was a multi-national longitudinal study of AD in Europe described elsewhere [17, 18]. It included both AD and age-matched control subjects with blood cell measures, neuropsychological assessments and gene expression data [19]. NINCDS-ADRDA criteria and Diagnostic and Statistical Manual of Mental Disorders (DSM-IV) were used to classify AD patients. Blood cell count measurements were generated at King’s College Hospital according to clinical standards for 285 of these subjects. For a subset of samples (*n* = 71), all these variables were available for two or more visits. Blood measure rate of change was calculated as the slope of a linear model using individual age at visit (years) with blood measure as the dependent variable. Namely, blood measure = *β*_0_ + *β*_1_ age + *ε* (where *β*_1_ is the slope used, *β*_0_ intercept and *ε* noise).

### Statistical analysis

#### UK Biobank

To test for associations between each cognitive function test and blood measure, we used a general linear model (GLM) per blood measure in which participants were filtered by age (> 60 years) to give a better comparison to AD patients. Cognitive function test was used as the outcome variable, and blood measure as the main exposure in each case. All *p* values were adjusted for multiple testing using Benjamini and Hochberg correction. A representative residual value for blood count was generated based on a linear model using device and acquisition route as covariants (FID: 30000-30284). This allowed correction for effects of blood collection method without impacting the cognitive function model. Demographic variables were also added as further covariates to correct for age, education, sex (FID: 31) and assessment centre (FID: 54) as described by Nevado–Holgado et al. [20]. Education level impacts on multiple outcome measures [21, 22], here, we included education within our model to adjust for socio-economic factors represented by schooling in different areas. However, we accept that education and cognition are correlated as people with stronger cognitive ability tend to stay in education longer and we have included education as a covariate assuming that as a generic adjustment of residual confounders, it will lead to conservative estimate of cognitive function. The same approach was used to test for the association between AD status and blood traits, with a GLM per blood measure including the same covariates as before. However, the population consisted of all participants older than 60 with a diagnosis of AD, plus a control participant (i.e. without AD) per case matched by age and gender. A representative residual value for blood count was generated based on a linear model using device and acquisition route as covariants (FID: 30000-30284). This allowed correction for effects of blood collection method without impacting the cognitive function model.

#### AddNeuroMed

To test for differences in the case and control sample sets, different statistical tests were applied depending on the number of available samples. An unpaired *t* test was used to assess for significant differences between the mean rates of change, while Kolmogorov–Smirnov test was used to discern a difference between the distributions of rates of change. *p* values were adjusted for false discovery rate in both instances. These simpler methods were required to capture differences in the case of a small sample set while, where sample size was large enough (for MMSE-tested patients), a GLM was applied instead with corrections for sex and age.

### Mendelian randomisation

The main genetic data analysis was based on the first released data batch of 152,736 participants from UK Biobank. Samples were filtered by ethnicity (FID: 22006, only keeping those with white genetic background); genetic sex (FID: 22001, removing those where stated gender did not match with real X–Y chromosome); related participants (FID: 22012, removing one from each common pair) and experimental checks (FID: 22050 and 22010) to leave 116,478 samples. A secondary replication analysis was performed on the interim set of genetic data (UK Biobank Release 2) which contained 335,423 participants. The dataset was processed following the method outlined by Bycroft et al. [23].

The SNPs for MR were selected based on two GWAS studies of blood traits with secondary validations as a filter [24, 25]. The SNP list was then filtered using the PhenomeScanner [26] tool to remove all SNPs with a known AD relationship, including SNPs located in the APOE/TOMM40 locus, to reduce the potential of pleiotropy errors. Remaining SNPs, with an info score > 0.9, were extracted from the imputed dataset. Subsets of SNPs specific to the blood measure were prepared to allow testing of instrument choice for pleiotropy. As blood measures are derived from common values, we selected three independent traits to study based on their association with the outcome variables: MCH; red blood cell distribution width (RDW) and reticylocyte count (RET). Association analysis was performed in SNPtest [27] for imputed data.

One-sample MR was implemented using the “Mendelian Randomisation” package from R [28] which incorporates three methods with different assumptions. The median weighted method or two-stage least squares estimation uses a median of the individual causal estimate per SNP, which is calculated from the ratio estimates of outcome’s regression coefficient divided by exposure [29]. The inverse-variance weighted (IVW) method uses the same ratio estimates but incorporates inverse-variance weights into the final summary estimate [30]. The Egger method is sensitive to SNP pleiotropy and allows the estimation of underlying bias by allowing a non-zero estimate for the intercept of the calculated ratio of beta values [31]. Comparing estimates from all of the methods shows the robustness of the overall analysis. Two-sample MR was performed with the “MRBase” R package [32] using the same instrument set.

### Gene expression analysis and pathway enrichment

RNA was extracted from blood samples and assayed on Illumina Human HT-12 Expression Beadchips, full details are described by Lunnon et al. [19]. While a subset of these samples was used for this analysis based on data completion, the full raw dataset is available as GEO DataSets with accession numbers GSE63060 and GSE63061. Two approaches were used for array expression analysis, LIMMA models were used for fold change calculations and the SAMr correlation method was used to generate permutated statistics for the patient based approach. Finally, the Kolmogorov–Smirnov test was used to evaluate KEGG pathways for significant enrichment. This pathway approach is described by Nevado–Holgado et al. [33] which, similar to GSEA, takes significance values from each individual gene and compares the overall distribution of expression rather than a simple binomial approach.

## Results

### Haemoglobin content has a significant association with cognitive function tests

**Fig. 1:**
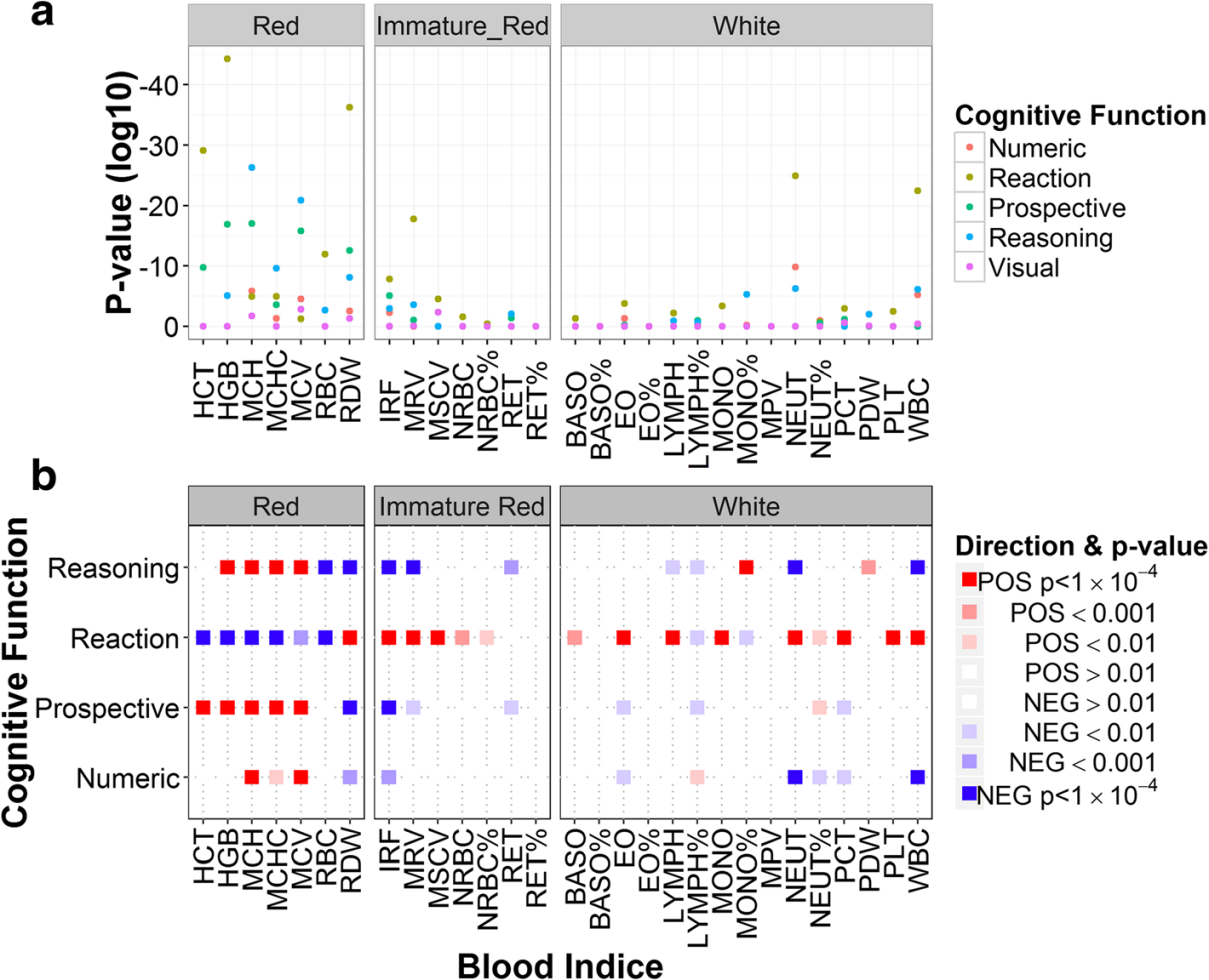
Cognitive tests have a significant effect on red blood cell measures. **a** There is a significant association between red blood cell measures and the reaction time, reasoning, numeric and prospective cognitive function tests. **b** Increased MCH and related indices have a positive effect on verbal–numeric reasoning, prospective and numeric memory (red squares). Reaction time is increased as haemoglobin decreases due to inverse nature of the reaction time test (blue squares). See Abbreviations for blood indices’ acronyms

Using the UK Biobank dataset, five cognitive function tests were compared to the complete blood cell indices set (Table 1). There was a significant association for red blood cell distribution width (RDW) and mean corpuscular haemoglobin (MCH) with outcomes on four cognitive tests including reaction time and verbal–numeric reasoning (Fig. 1a). Although reaction time was associated with white cell count and neutrophil number, associations with red cell indices were considerably more extensive.

**Table 1:** Associations between blood traits and cognitive function tests as revealed by linear modelling

	Numeric beta	Numeric *p*	Reaction beta	Reaction *p*	Prospective beta	Prospective *p*	Reasoning beta	Reasoning p	Visual beta	Visual *p*
Red										
RBC	− 0.085	0.019	− 0.012	*8.86 × 10* ^*−14*^	0.011	0.642	− 0.089	2.39 × 10^*−5*^	− 0.013	0.063
HGB	0.026	0.056	− 0.009	*6.67 × 10* ^*−46*^	0.076	*1.44 × 10* ^*−18*^	0.040	*2.26 × 10* ^*−7*^	0.0004	0.884
HCT	0.002	0.701	− 0.002	*8.78 × 10* ^*−31*^	0.020	*6.97 × 10* ^*−12*^	0.005	0.045	0.0001	0.884
MCV	0.015	*7.12 × 10* ^*−7*^	− 0.001	4.08 × 10^−4^	0.017	*2.29 × 10* ^*−17*^	0.017	*1.64 × 10* ^*−22*^	0.002	3.15 × 10^−4^
MCH	0.036	*1.53 × 10* ^*−7*^	− 0.002	*8.45 × 10* ^*−8*^	0.042	*1.44 × 10* ^*−18*^	0.044	*1.92 × 10* ^*−28*^	0.004	0.001
MCHC	0.033	0.004	− 0.003	*7.94 × 10* ^*−8*^	0.041	1.15 × 10^−5^	0.048	*7.33 × 10* ^*−12*^	0.001	0.787
RDW	− 0.055	1.27 × 10^−4^	0.009	*1.23 × 10* ^*−38*^	− 0.070	*1.71 × 10* ^*−14*^	− 0.053	*3.06 × 10* ^*− 10*^	− 0.008	0.003
Immature red										
RET%	− 0.016	0.442	0.001	0.227	− 0.021	0.100	− 0.018	0.104	− 0.001	0.812
RET	− 1.346	0.025	− 0.001	0.984	− 1.341	0.001	− 1.301	9.40 × 10^−5^	− 0.067	0.375
MRV	− 0.002	0.432	0.001	*6.34 × 10* ^*−21*^	− 0.003	0.009	− 0.005	*7.18 × 10* ^*−6*^	0.001	0.022
MSCV	0.002	0.512	0.001	*2.58 × 10* ^*−7*^	0.001	0.642	− 0.001	0.413	0.002	4.24 × 10^−4^
IRF	− 0.857	1.41 × 10^−4^	0.066	*3.70 × 10* ^*−10*^	− 0.784	*4.05 × 10* ^*−7*^	− 0.544	5.97 × 10^−5^	− 0.077	0.083
NRBC	− 0.186	0.799	0.072	2.67 × 10^−4^	− 0.404	0.227	− 0.385	0.241	0.026	0.879
NRBC%	− 0.003	0.945	0.005	0.004	− 0.021	0.326	− 0.020	0.352	− 0.001	0.884
White										
WBC	− 0.032	*4.47 × 10* ^*−7*^	0.003	*2.16 × 10* ^*−24*^	− 0.010	0.020	− 0.020	*2.06 × 10* ^*−8*^	− 0.003	0.013
PLT	− 0.0004	0.071	0.00004	1.53 × 10^−4^	0.000	0.020	0.000	0.825	0.000	0.273
PCT	− 0.802	0.008	0.053	6.28 × 10^−5^	− 0.668	0.001	− 0.187	0.344	− 0.142	0.010
MPV	− 0.016	0.214	− 0.00001	0.984	− 0.012	0.163	− 0.015	0.045	− 0.004	0.083
PDW	0.007	0.862	− 0.002	0.054	0.035	0.054	0.056	0.000	− 0.011	0.022
LYMPH	− 0.001	0.945	0.002	8.65 × 10^−5^	− 0.011	0.109	− 0.018	0.002	− 0.003	0.155
MONO	− 0.087	0.118	0.013	*5.28 × 10* ^*−6*^	− 0.041	0.241	0.027	0.420	− 0.011	0.390
NEUT	− 0.061	*3.15 × 10* ^*−11*^	0.005	*1.77 × 10* ^*−26*^	− 0.009	0.155	− 0.031	*1.90 × 10* ^*−8*^	− 0.004	0.036
EO	− 0.318	0.001	0.021	*3.18 × 10* ^*−6*^	− 0.175	0.010	− 0.005	0.927	− 0.024	0.233
BASO	− 0.010	0.973	0.046	3.61 × 10^−4^	− 0.498	0.014	− 0.025	0.910	− 0.007	0.884
LYMPH%	0.005	0.009	− 0.0002	0.004246	− 0.004	0.002	− 0.003	0.003	− 0.0004	0.232
MONO%	0.012	0.011	− 0.001	0.012	0.003	0.326	0.013	*1.44 × 10* ^*−7*^	0.0001	0.884
NEUT%	− 0.005	0.003	0.0002	0.003	0.003	0.005	0.0002	0.910	0.0003	0.294
EO%	− 0.005	0.593	0.0003	0.316	− 0.009	0.085	0.011	0.013	− 0.0004	0.884
BASO%	0.037	0.186	0.002	0.126	− 0.030	0.056	0.022	0.140	0.002	0.787

Highly significant results are marked with italic fonts (*p* < 10^−5^). See Abbreviations section for acronyms

Performance on the reasoning test was positively correlated with red blood cell haemoglobin (Fig. 1b). Haemoglobin concentration (HGB), MCH and mean corpuscular haemoglobin concentration (MCHC) were higher in participants with higher reasoning scores (beta = 0.04, 0.04, 0.05 and *p* value = 2.26 × 10^−7^, 1.92 × 10^−28^, 7.33 × 10^−12^ respectively). The same correlation trend is seen in the numeric and prospective memory tests. Reaction time was inversely associated with HGB, MCH and MCHC measures (beta = − 0.009, − 0.003, − 0.002 and *p* value = 6.67 × 10^−46^, 7.94 × 10^−8^, 8.45 × 10^−8^ respectively); reflecting the same direction of change as with other cognition measures as increased reaction time is reflective of relatively worse cognition. We found that RDW was inversely correlated with four tests of cognitive function (beta between − 0.053 and − 0.008, *p* value from 1.71 × 10^−14^ to 0.003).

Interestingly, the reticulocyte (RET) measures, although highly variable, show the largest significant beta scores (beta between − 1.34 and − 1.310 with *p* values from 0.025 to 9.4 × 10^−5^). As these sets of measures are used clinically to diagnose iron-deficient anaemia, we estimated the proportion of participants with anaemia according to NICE guidelines and repeated the analysis. Participants with anaemia, so defined, had a significant reduction in performance on cognitive tests for three measures (prospective, numeric and reasoning) and increased reaction time score (*p* < 0.0005, Additional file 2: Figure S1).

### Mean corpuscular haemoglobin and red blood cell distribution width have a causative relationship with verbal–numeric reasoning

**Fig. 2:**
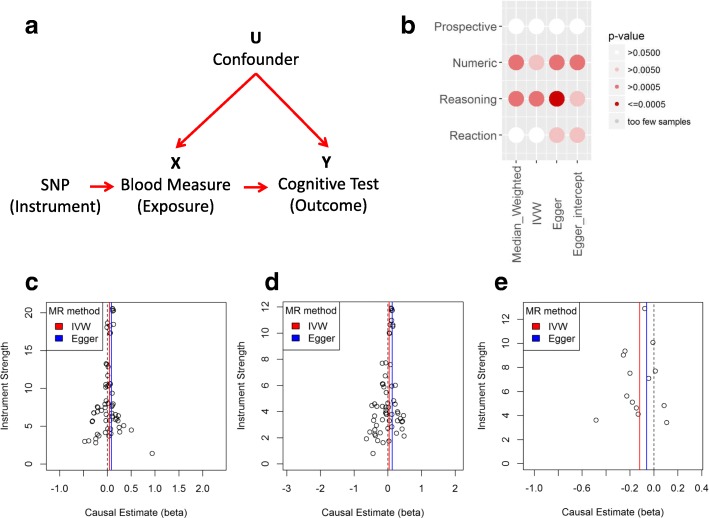
MCH has a significant effect on the reasoning cognition in multiple MR analysis approaches. **a** Mendelian randomisation model used for analysis. **b** *p* values are significant (> 0.005) in multiple MR methods for the MCH measure (exposure) in the reasoning and numeric traits. Significance in more than one test method is important to rule out pleiotropy among instruments. **c** MCH instrument (SNP) causal estimates for the reasoning (outcome) show symmetry about 0 indicating a robust analysis (without pleiotropy). **d** MCH instrument causal estimates for the numeric trait. **e** Instrument causal estimates for the reasoning trait compared to RDW

Using UK Biobank to estimate a direction of effect, we applied a single-sample MR model where the cognitive test was the outcome variable, the blood measure the mediating exposure variable, and SNPs known to be related to the blood measure were used as instruments (Fig. 2a). In all cases, we used three alternative MR methods to discount the possibility of pleiotropy among SNPs (Table 2) as well as plots to assess SNP beta scores (Fig. 2c–e). This approach identified a significant effect on the numeric and reasoning traits from the MCH measure (Fig. 2b). The effect between MCH and reasoning traits was replicated in an analysis using in the interim release of the full UK Biobank genetic data where we were able to reproduce the same direction of effect (Additional file 1: Table S2). In addition, two-sample MR was used to analyse the association in an alternative sample set (Additional file 2: Figure S2). The UK Biobank cognitive reasoning was used as the outcome, and MCH beta scores from the MRBase library were introduced as the new exposure to duplicate the significant results shown in our main one-sample results (*p* values < 0.05 for all three MR methods).

**Table 2:** Associations from MCH and RDW to cognitive tests as revealed by MR

	Median weighted beta [CI]	Median weighted *p*	IVW beta [CI]	IVW *p*	Egger beta [CI]	Egger *p*
MCH trait						
Reaction	0.0199 [− 0.0005, 0.0404]	0.0562	0.0091 [− 0.0057, 0.0238]	0.2320	*0.0363 [0.01, 0.0627]*	*0.0069*
Reasoning	*0.0568 [0.0195, 0.0941]*	*0.0028*	*0.0458 [0.0194, 0.0722]*	*0.0012*	*0.0871 [0.0392, 0.1351]*	*0.0004*
Numeric	*0.0977 [0.0364, 0.159]*	*0.0018*	*0.0443 [0.0021, 0.0864]*	*0.0392*	*0.1327 [0.0544, 0.2111]*	*0.0009*
Visual	− 0.0015 [− 0.0217, 0.0188]	0.8855	− 0.0097 [− 0.0235, 0.0041]	0.1521	− 0.0182 [− 0.0439, 0.0074]	0.1640
Prospective	0.0706 [− 0.0149, 0.1562]	0.1055	0.0311 [− 0.0289, 0.0912]	0.3075	0.0506 [− 0.0612, 0.1623]	0.3752
RDW trait						
Reaction	− 0.0245 [− 0.0788, 0.0298]	0.3758	− 0.0135 [− 0.0541, 0.0271]	0.4528	− 0.0294 [− 0.1284, 0.0696]	0.5601
Reasoning	*− 0.1036 [− 0.2041, − 0.0031]*	*0.0432*	*− 0.1191 [− 0.1907, − 0.0474]*	*0.0025*	− 0.0602 [− 0.235, 0.1146]	0.4996
Numeric	*− 0.2121 [− 0.3769, − 0.0472]*	*0.0117*	− 0.111 [− 0.2345, 0.0124]	0.0714	*− 0.3243 [− 0.6247, − 0.0238]*	*0.0344*
Visual	0.0086 [− 0.0501, 0.0673]	0.7739	0.0428 [− 0.0092, 0.0947]	0.1307	0.0708 [− 0.0598, 0.2015]	0.2880
Prospective	− 0.1024 [− 0.3368, 0.132]	0.3917	− 0.1108 [− 0.287, 0.0654]	0.1858	0.0278 [− 0.4019, 0.4575]	0.8992

Significant results are marked with italic fonts (*p* < 0.05). Higher and lower confidence intervals for beta scores given in square brackets [CI]

RDW also showed significant effects in several of the MR tests for the reasoning and numeric traits (Table 2). Beta scores were negative suggesting an inverse relationship whereby RDW decreases as the cognition improves (Fig. 2e). Given the relationship between haemoglobin measures and cognitive tests, red blood cell indices were selected based on GLM results (Table 1), and their unique derivation source, to fit independent testing assumptions. MCH and RDW were the best candidates based on results from analyses with cognitive tests and imply that both haemoglobin levels and red blood cells themselves have a potentially causative effect on cognition (Table 2). RET was included as it is an independent measure with strong beta scores but was not significant (Additional file 1: Table S3).

### Changes in red blood indices are also associated with Alzheimer’s disease

UK Biobank participants gave consent for linkage to medical records and using Hospital Episodes Statistics data a subset of participants with a recorded clinical diagnosis of AD or other dementia was identified using ICD10 codes. This subset was then age and gender matched to a control group (*n* = 1170). Using this sub-cohort anaemia was found to be significantly increased in people with AD (beta = 0.26, *p* value = 2.3 × 10^−6^) and the RBC and HGB indices were all decreased in the AD participant set (beta = − 0.66 and − 0.18 respectively, adjusted *p* values < 0.05; Additional file 1: Table S4).

### Replication of the red blood cell association in an independent cohort

**Fig. 3:**
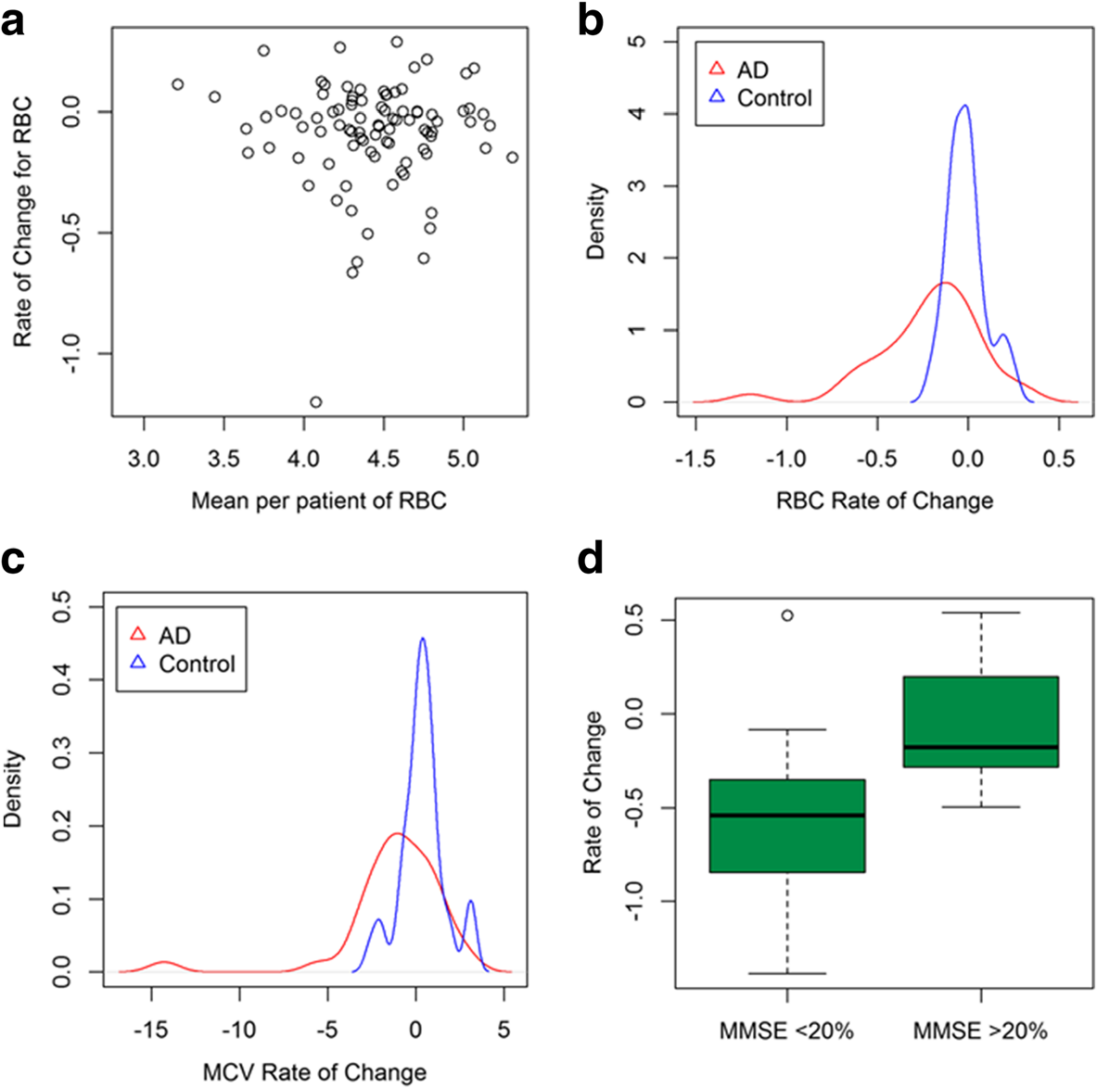
Rate of change in red blood cells emphasises differences in AD case–control samples. **a** Rate of change per patient is not correlated with mean per patient. **b** The distribution of RBC is significantly decreased in AD compared to controls. **c** The distribution of MCV, a haemoglobin measure, is significantly decreased in AD patients. **d** RBC rate of change is significantly different for high and low MMSE scores

We then turned to the AddNeuroMed cohort to replicate these findings using complementary analyses. We determined rate of change measures per participant to incorporate multiple visit data when the participant made at least three visits between age of patient at visit (years) and each cell count measure (Additional file 2: Figure S3). These rate of change values were not correlated to the mean statistic (rho = − 0.031, Fig. 3a) suggesting that they provide additional information over and above the mean. We found a significant difference between AD case and normal cognition control subjects in five red blood cell rate of change measures (*p* value < 0.05, Table 3). A decline in rate of change was shown in the AD cases compared to control patients, with Fig. 3b, c showing the difference in distributions between RBC (*p* value = 2.21 × 10^− 4^) and mean corpuscular volume (MCV, *p* value = 1.95 × 10^−3^). The test was repeated using the MMSE scores per patient as an assessment of cognition. Using the highest and lowest scores (± 20%) and despite the low sample numbers (*n* = 53) and therefore lack of power, a significant difference remained (adjusted *p* value < 0.005) in three red blood cell rate of change measures between low and high MMSE (Fig. 3d). Finally, using MMSE as a continuous measure in a linear model, significant association was shown between MMSE score and four red blood cell measures including MCH (Table 3).

**Table 3:** Significant Differences for red blood cell measures in an independent sample set

	AD case–control	AD case–control	MMSE			
	*T* test	KS test	*T* test	KS test	Beta	*p* value
Red						
Red blood cell count (RBC)	*2.65 × 10* ^*−03*^	*2.21 × 10* ^*−04*^	*4.06 × 10* ^*−04*^	*5.63 × 10* ^*−04*^	*0.02*	*7.04 × 10* ^*−03*^
Haemoglobin (HGB)	*0.036*	*1.92 × 10* ^*−03*^	*4.06 × 10* ^*−04*^	*2.25 × 10* ^*−04*^	*0.04*	*0.036*
Packed cell volume (PCV)	*9.42 × 10* ^*−04*^	*1.61 × 10* ^*−06*^	*4.06 × 10* ^*−04*^	*2.25 × 10* ^*−04*^	*0.002*	*0.026*
Mean corpuscular volume (MCV)	*0.033*	*1.95 × 10* ^*−03*^	0.483	0.092	0.01	0.929
Mean corpuscular haemoglobin (MCH)	*0.036*	*1.92 × 10* ^*−03*^	0.244	0.054	*− 0.05*	*0.027*
Red cell distribution width (RDW)	0.103	0.142	0.609	0.606	0.01	0.868
White						
Platelet (PLT)	*0.036*	*1.55 × 10* ^*−03*^	0.244	0.092	− 1.18	0.369
Platelet distribution width (PDW)	*0.036*	0.142	0.244	0.606	− 0.36	0.369
White blood cell (WBC)	0.382	0.345	0.944	0.609	0.05	0.929
Neutrophils	0.729	0.105	0.992	0.487	− 0.02	0.914
Lymphocytes	0.867	0.379	0.944	0.154	0.00	0.929
Monocytes	0.867	0.379	0.944	0.606	0.00	0.914
Eosinophils	0.867	*0.030*	0.944	0.361	0.00	0.369
Basophils	0.382	0.105	0.449	0.065	0.00	0.127

The AddNeuroMed dataset was divided by two variables for testing. Cases and control groups were compared by *t* test and Kolmogorov–Smirnov (KS) tests. MMSE was used to split samples into two groups for *t* and Kolmogorov–Smirnov (KS) tests. Results using MMSE from all samples using a linear model are also shown (beta estimate and *p* value given). Significant results are marked with italic fonts (*p* < 0.05)

### Pathway enrichment analysis indicates changes in MCH may have an impact on haematological gene expression

As the AddNeuroMed cohort also contained whole blood whole genome transcript data, we were able to use this dataset to explore, using several approaches, the gene expression patterns and hence KEGG pathways, linked to both blood traits and to AD. Initially, we used all subjects with both expression and rate of change in MCH data in a fold change analysis to look for significantly associated genes (37 patients), finding an enrichment for the glycosylphosphatidylinositol (GPI) anchor biosynthesis pathway (*p* value = 0.0107) in those with greatest rate of change in MCH. Defects in this pathway cause paroxysmal nocturnal haemoglobinuria, a genetic disorder whereby the immune system destroys red blood cells. We then focussed in on the AD group with complete data as above (*n* = 22) to look for correlation between rate of decline in blood indices and gene expression. Using this filtered approach, we detected an enrichment for haematopoietic cell linage pathway correlating with MCH rate of decline (*p* value = 0.0088, Additional file 1: Table S5). In both cases, we found weaker *p* values at the initial analysis stage, which is to be expected given the sample size.

## Discussion

Recently, increasing attention is being paid, with considerable justification, to environmental factors that might influence the development of dementia. As pharmacological strategies for prevention have not yet yielded success and as the number of people with dementia continues to rise then modifying environmental factors to reduce incidence of dementia is an increasingly attractive prospect. Supportive evidence for such an approach has come from multiple lines of evidence that despite increasing prevalence, the incidence of dementia might be falling; an observation that might be due to improved modification of cardiovascular risk factors. However, other non-genetic risk factors derived from observational study cannot be assumed to be causative and because of this, modification may not prove to be successful in reducing further the incidence of dementia. It becomes therefore, of paramount importance to determine causality, including through the use of MR techniques. However, previously, this approach has offered relatively little support to the hypothesis that modification of environmental risk factors such as LDL cholesterol, glycemic traits, diabetes, body mass index or education would reduce incidence of dementia [34]. In fact, counter intuitively, Ostergaard et al. [35] find *higher* systolic blood pressure to be associated with *decreased* risk of dementia, suggesting either that blood pressure has opposite effects on risk of dementia and of cardiovascular disease or that another factor associated with hypertension, most obviously anti-hypertensive medication, has a protective effect. There is therefore an evidence gap at present between observational studies proposing risk factors for modification and robust proof of concept for such modification including causality. Without this evidence, the only approach is to perform an interventional study of environmental modification, a challenge given the difficulties and costs of such public health measures. Evidence from approaches such as MR for causality would add considerably to the justification for such interventional studies.

We present evidence here for a primary causative association between indices indicative of relatively poor red cell function and cognitive function and, using MR with genetic loci previously found to have robust relationship with red cell phenotypes, findings that strongly suggest that lower haemoglobin has a causal impact on cognitive performance. Moreover, secondary analyses are in line with previous findings showing an association between anaemia and meeting operationalised criteria is a risk factor for dementia as well as lower cognition. Specifically, in UK Biobank data, we find lower MCH and RDW to be associated with relatively lower verbal–numeric reasoning and numeric memory and that measures indicative of anaemia, or a clinical diagnosis of anaemia, are associated with decreased cognitive function. This result replicates findings in a larger healthy population (*n* > 37,000) compared to previous studies [11, 14]. In complementary analyses in AddNeuroMed, a cohort study of dementia, we similarly find that red blood cell indices including red cell count, PCV and HGB are associated with AD and with decline in cognitive function measures. Using genetic loci strongly associated with these blood traits, we find associations with poorer cognitive function strongly suggesting a causative relationship with cognitive performance and by implication with dementia. Finally, pathway analysis of gene expression in blood in the AddNeuroMed cohort finds genes known to be linked with anaemia and the pathway of haematopoietic cell linage to be associated with changes in red cell indices adding further to the weight of evidence suggesting that these observations are indicative of true biological association.

The RBC indices we observe to be most strongly associated with cognitive outcomes are MCH and RDW, measures commonly associated with iron deficiency anaemia [36] indicating a possible deficit in haem synthesis or iron metabolism as an underlying trait. A possible relationship between neurodegeneration and iron has been investigated in other MR studies. Pichler et al. [37] used MR with three SNP instruments to find that increased iron reduces the risk of Parkinson’s disease and implying that there may well be a causal association in other similar diseases. However, Lupton et al. [38] used genetic determinants of the serum iron measures transferrin and ferritin in a reanalysis of large-scale GWAS data but found no association with AD. One possible explanation for this apparent discrepancy is the use of MCH in the present study, reportedly a more reliable measure of haemoglobin not influenced by sample storage conditions or cell counter methods [36]. Another potential explanation is the difference in instrument choice available from comprehensive GWAS studies of the blood indices [39]. By approaching the problem from the opposite direction using known genetic blood traits, we were able to detect a significant link not seen using AD genetics. The complexities of relationship between iron and AD have been shown using other experimental methods. For example, iron metabolism is disrupted in cortical neurons and the beta-amyloid protein precursor has ferroxidase activity in mouse models [40]. Telling et al. [41] have described a correlation between iron biochemistry and amyloid beta. These results show the relationship at the molecular level and may indicate a potential mechanism for iron within AD. The relevance of blood indices to the iron deposition has been shown in other UK Biobank based studies. Miller et al. [42] showed a correlation between the blood indices and T2* image derived phenotypes from the brain scans (which reflects iron deposition). In addition, a recent GWAS study showed significant associations between the T2* subcortical regions and genes related to iron transport such as *TF*, *HFE* and *SLC25A37* [43].

We recognise that there are limitations to this study. The five cognitive tests were generally in agreement; however, there was some discrepancy in the visual memory task. The task itself involved matching of pairs and although the mismatched score was used to improve reliability of the testing measure there are still weaknesses in this data set. Other studies have shown the measure has a low reliability score of 0.15 [16] and potential weaknesses of test method may be impacting on our own analysis results. The main inference for the MR analysis is use of cognitive performance as a proxy representative for AD. An alternative would have been to use the AD phenotype as the mediating exposure, but the low number of AD patients recorded in UK Biobank seriously limits the statistical sensitivity of this approach. Additionally, this only had borderline significance in other studies [44].

Pleiotropy of instruments is a common limitation of MR approaches. We used a number of tests to check for pleiotropy effects on the results including Egger methods and confirmational plots.

Using the rate of change statistic from the blood measures, we were able to determine a difference between AD patients and controls. This is not a standard approach, possibly due to limited data available for multiple visits; however, it was very informative. We found differences that were reproduced in a larger set that were not detected otherwise. Using the same dataset but taking a mean statistic per patient, rather than time decline, we detected a difference in white blood cell measure for basophils [45]. Given the known effects of AD on blood measures, it seems likely that both blood types are affected. Nonetheless, both methods warrant replication in a larger, independent dataset. We have also presented some interesting pathway enrichment results yielding pathways which warrant replication in an independent sample set with the goal of identifying related genes.

## Conclusions

We have presented here further evidence for the association between red blood cell measures normally indicative of anaemia and measures of both poor cognitive performance and of dementia. Using a robust MR approach, we are able to determine that this relationship is one of causality and not consequence suggesting that reversing these changes might slow or prevent the onset of dementia. These findings require replication in other datasets but already derive from one very large and one very detailed cohort study. If they are replicated then the implications are considerable. As our findings apply to people with decreased cognitive function within the normal range as well as to people with established dementia then the implication is that the causal relationship between decreased red cell function and anaemia are an early, preclinical influence on disease that continues through to the dementia syndrome. It follows that measures to reduce or reverse poor red cell function might be both preventative and therapeutic at least in part. If this was proven in interventional studies then such screening measures, already in widespread use in the population, might be used to identify people for these and indeed for other secondary prevention interventions as they become available.

## Additional files

Additional file 1: **Table S1.** Sample set description. **Table S2.** MR replication in release 2 of the UK Biobank data. **Table S3.** MR results for RET trait. **Table S4.** General linear model results show an association between AD patients and red blood indices in UK Biobank. **Table S5.** Significantly enriched KEGG pathways for MCH decline correlated genes. (XLSX 18 kb)

Additional file 2: **Figure S1.** Anaemia has a significant effect on four cognitive test measures. **Figure S2.** Two sample MR results replicating the direction of effect for MCH on the verbal–numeric reasoning outcome. **Figure S3.** Rate of change as a measure of red blood cell count. (PDF 371 kb)

## Abbreviations

AD: Alzheimer’s disease
BASO: Basophill count
BASO%: Basophill percentage
EO: Eosinophill count
EO%: Eosinophill percentage
HCT: Haematocrit percentage
HGB: Haemoglobin concentration
IRF: Immature reticulocyte fraction
LYMPH: Lymphocyte count
LYMPH%: Lymphocyte percentage
MCH: Mean corpuscular haemoglobin
MCHC: Mean corpuscular haemoglobin concentration
MCV: Mean corpuscular volume
MONO: Monocyte count
MONO%: Monocyte percentage
MPV: Mean platelet volume
MR: Mendelian randomisation
MRV: Mean reticulocyte volume
MSCV: Mean sphered cell volume
NEUT: Neutrophill count
NEUT%: Neutrophill percentage
NRBC: Nucleated red blood cell count
NRBC%: Nucleated red blood cell percentage
PCT: Platelet crit
PCV: Packed cell volume
PDW: Platelet distribution width
PLT: Platelet count
RBC: Red blood cell count
RDW: Red blood cell distribution width
RET: Reticulocyte count
RET%: Reticulocyte percentage
WBC: White blood cell count
