# Supplementary material for: Red blood cell indices and anaemia as causative factors for cognitive function deficits and for Alzheimer’s disease
Source: Genome Med. 2018 Jun 28;10:51. doi: 10.1186/s13073-018-0556-z (PMC6022699; doi:10.1186/s13073-018-0556-z)

Additional file 2: Fig. S1- Anaemia has a significant effect on four cognitive test measures

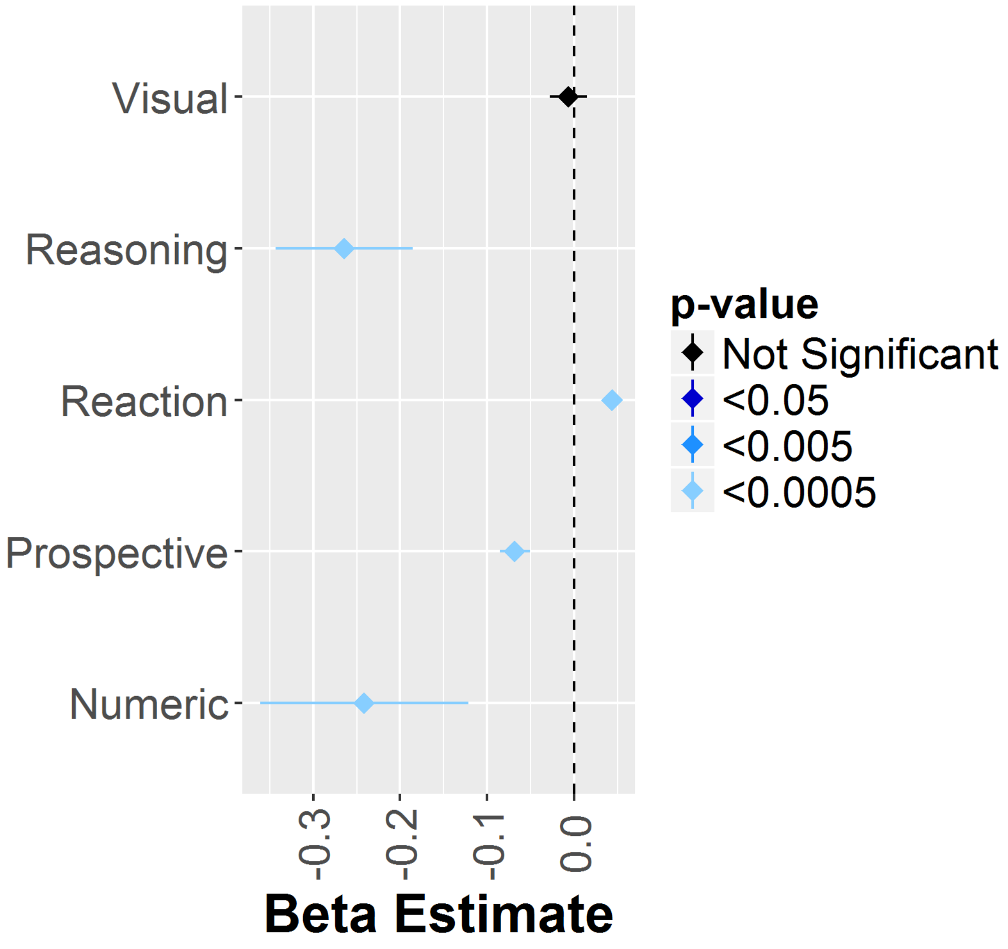

Additional file 2: Fig. S2 - 2 sample MR Results replicating the direction of effect for MCH on the Verbal-Numeric Reasoning outcome

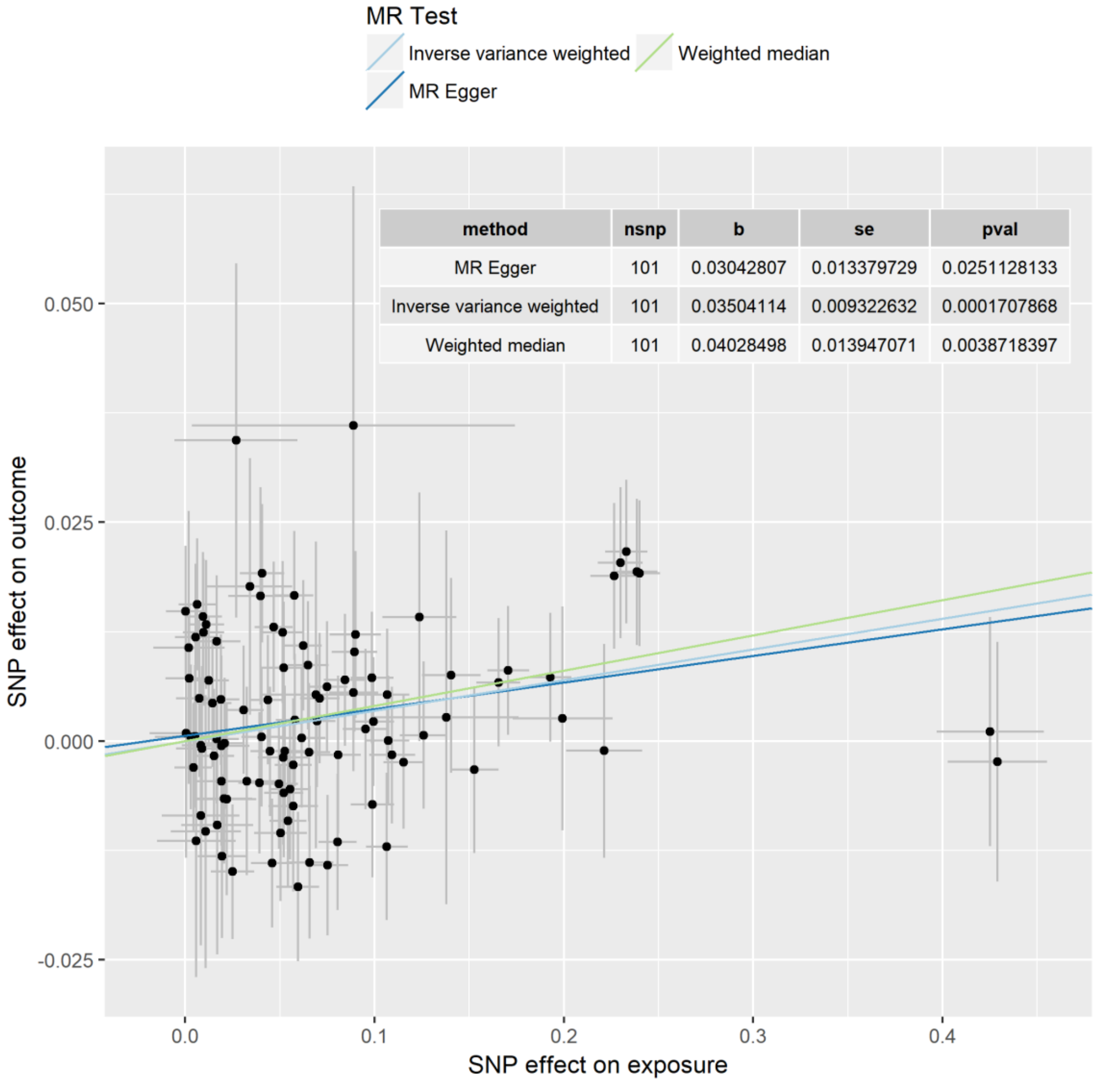

method	nsnp	b	se	pval
MR Egger	101	0.03042807	0.013379729	0.0251128133
Inverse variance weighted	101	0.03504114	0.009322632	0.0001707868
Weighted median	101	0.04028498	0.013947071	0.0038718397

Additional file 2: Fig. S3 - Rate of Change as a measure of red blood cell count

Blood cell count is shown compared to visit age showing the time between visits for both cases and controls.

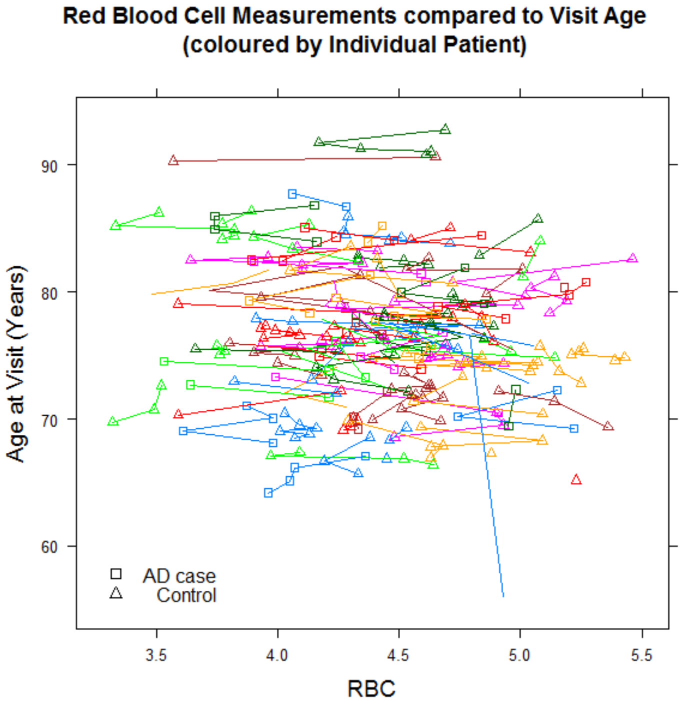

Supplement: Supplementary file 2 — Figure S1. Anaemia has a significant effect on four cognitive test measures. Figure S2. Two sample MR results replicating the direction of effect for MCH on the verbal–numeric reasoning outcome. Figure S3. Rate of change as a measure of red blood cell count. (PDF 371 kb) [file 13073_2018_556_MOESM2_ESM.pdf]
